# Feasibility of Using Carvacrol/Starch Edible Coatings to Improve the Quality of Paipa Cheese

**DOI:** 10.3390/polym13152516

**Published:** 2021-07-30

**Authors:** Alex López-Córdoba

**Affiliations:** Grupo de Investigación en Bioeconomía y Sostenibilidad Agroalimentaria, Facultad Seccional Duitama, Escuela de Administración de Empresas Agropecuarias, Universidad Pedagógica y Tecnológica de Colombia, Carrera 18 con Calle 22 Duitama, Boyaca 150461, Colombia; alex.lopez01@uptc.edu.co; Tel.: +57-8-7604100

**Keywords:** artisanal cheese, dairy products, polymer applications, protected geographical indication

## Abstract

Paipa cheese is the only Colombian semi-ripened cheese with protected geographical indication. In the current work, the effect of applying starch coatings carrying carvacrol on Paipa cheeses was analyzed. Coatings were prepared based on blends of potato starch (2 g/100 g), carvacrol (0.1 g/100 g), polysorbate 80, glycerol, and water and applied to the cheese’s surface by brushing. Uncoated cheeses were also analyzed for comparison. Moreover, films were prepared and characterized in terms of their moisture content, water vapor permeability, mechanical properties, transparency, water solubility, swelling (%), and antioxidant activity. Carvacrol/starch films showed a slight decrease in their water solubility and Young’s modulus, while not significant changes were observed in water vapor permeability, moisture content, transparency, and swelling behavior, in comparison with the starch films. After application on the Paipa cheeses, the carvacrol/starch coatings enhanced the brightness of the cheeses without causing significant changes in water activity, moisture content, color attributes, and mesophilic aerobic bacteria and molds/yeasts count. Moreover, edible coatings have a significant effect on the hardness, the gumminess, the springiness, and the chewiness of the Paipa cheese. Coated cheeses were better preserved at day 60 of storage because they did not show changes in their lightness, hardness, and springiness.

## 1. Introduction

Paipa cheese is a medium fat, semi-ripened hard cheese manufactured from raw cow’s milk by enzymatic coagulation which has received protected geographical indication (PGI) by Colombian regulations [1]. This artisanal cheese is popular in the Colombian market because of its ancestral production practices, nutritional properties, and particular and distinctive flavor and texture.

Paipa cheese is made mainly by farmers in the municipalities of Paipa and Sotaquirá (i.e., the Eastern Ranges of the Colombian Andes), based on traditional production processes, in which the milk is coagulated enzymatically and then the curds are kneaded and pressed into round molds manually. The cheese is allowed to ripen at ambient conditions (15–20 °C/RH: 65–70%) around 21 days and then commercialized, commonly without any packaging. There are very few cheesemakers using an industrial production process that commercialized Paipa cheese in synthetic polymer-based packaging, with and without vacuum or modified atmospheres. However, it is well known that yeast and bacteria cheese spoilage could take place even with at low oxygen concentration [2,3]. Moreover, once the packaging is opened, microbial cheese contamination needs to be prevented.

Currently, the quality of Paipa cheese is mainly limited by appearance and textural changes during storage. Lactic acid bacteria and other bacterial groups, including potential spoilage, toxinogenic or pathogenic bacteria can also be present during ripening and in the finished product [1]. Therefore, there is a significant interest to develop complementary cheese preservation methods to improve the food safety of Paipa cheese.

Edible coatings constitute a useful strategy to prevent the spoilage of cheese products because they act as a selective barrier, decreasing the water vapor and the gases exchange between the food and the surrounding environment, enhance the visual appearance and prevent the occurrence of lipid oxidation reactions [4,5]. Several researchers have studied the effect of the application of edible coatings based on natural polymers (e.g., chitosan, guar gum, alginate, starch and whey protein) on the shelf life of different types of cheeses [6,7]. In particular, in the case of semi- or ripened cheese, it has been reported that the coatings prevent the water loss and protect the cheese from microbial contamination when they are applied during ripening [5]. In addition, when the coating is applied at the end of the ripening period, it protects the cheese from physical damage during transport and distribution and/or to give the cheese a specific appearance.

Starch is a well-known film-forming natural polymer that produce transparent, odorless, tasteless, and colorless edible films and coatings [8]. Some researchers have developed edible coatings based on blends of starch with other natural polymers and/or food-grade additives for application on cheeses [9,10]. Berti et al., 2020 reported that the use of starch coatings reinforced with rice bran and containing nisin and natamycin was a useful strategy to prevent the post-process and external contamination of Argentinian Port Salut cheese [11].

Essential oils and oil compounds are considered to be effective antimicrobial and antioxidant agents. In particular, the carvacrol (i.e., an isomeric monoterpenoid) has proven to inhibit both Gram-positive and Gram-negative bacteria growth in several food products [12]. There are few studies that deal with the effect of carvacrol-containing edible coatings on the preservation of cheeses. Kuorwel et al., 2014 reported that the application of starch-based coatings carrying linalool, carvacrol and thymol on the Cheddar cheese surface produced an antifungal effect against *Aspergillus niger* [13].

In the current work, the effect of the application of a new starch edible coating carrying carvacrol on the physicochemical and microbiological properties of Paipa cheese was evaluated. These characteristics were compared with the uncoated Paipa cheese at the initial time and after 60 days of storage. To the best of the author’s knowledge, this is the first time that a study about the effect of edible coatings on the quality of Paipa cheese during storage is reported.

## 2. Materials and Methods

### 2.1. Materials

Potato starch was isolated from fresh potato tubers variety Diacol Capiro according to Doporto et al. [14]. Briefly, potatoes were washed, sanitized (250 ppm of chlorine, 10 min), peeled and pulped with a grater. The grated potatoes were blended with water (2 L water/kg) and stored at 4 °C for 24 h. The blend was filtered using a cheesecloth, and the starch slurry was decanted at 4 °C. The supernatant was discarded, and the starch cake was recovered, dried at 40 °C for 14 h in a hot-air oven, and milled. Figure S1 shows an image of the starch granules obtained by scanning electron microscopy. Besides, some characteristics of the isolated starch are shown in Table S1.

Carvacrol (98% purity) and sodium hydroxide were purchased from Sigma Aldrich (Sigma Aldrich Inc., St. Louis, MO, USA). Glycerol was purchased from J. T. Baker (J. T. Baker Inc., New Jersey, NJ, USA) and Polysorbate 80 was purchased from Loba Chemie (Loba Chemie Inc., Mumbai, India). All other chemicals used were also of analytical grade.

### 2.2. Preparation of Coatings and Films

Carvacrol/starch edible coatings were produced as reported in previous works [8,15]. Briefly, blends containing potato starch (2 g/100 g), glycerol (0.6 g/100 g) and distilled water (97.4 g/100 g) were prepared and heated until 93 °C under constant stirring. After cooling, carvacrol (0.1 g/100 g of blend) and Polysorbate 80 (0.01 g/100 g of blend) were added to the starch suspension and the mixture was homogenized at 1000 rpm for 10 min using a vertical agitator IKA C- MAG HS4 (IKA^®^ WERKE, Strufen, Germany) and then degassed using a vacuum pump. The coating formulation was chosen based on the results of previous work and preliminary experiments [8,15,16]. In particular, the carvacrol concentration was selected considering the results of preliminary sensory assays and the study reported by Kuorwel [13] about the antifungal activity of starch-based coatings carrying linalool, carvacrol and thymol on the Cheddar cheese.

Films made with the coating formulation were characterized [15]. Carvacrol/starch coating solution was poured into polypropylene plates and dried at 50 °C for 24 h. Then, dried films were peeled from the plates and conditioned at room temperature into desiccators containing a supersaturated solution of sodium bromide (RH~57%) for 48 h. Films were characterized in terms of their moisture content, water vapor permeability, mechanical properties, transparency, water solubility, swelling (%), and antioxidant activity following the optimized protocols described in previous works [8,15]. Starch films without carvacrol were also fabricated and characterized as mentioned above for comparison.

### 2.3. Application of Coatings

The Paipa cheeses were kindly donated by a local formal producer (Lácteos Campo Real^®^, Sotaquirá, Colombia). These were made following the standard procedure described in the Colombian regulations for the protected denomination of the origin of the cheese. The ripening was performed on wooden shelves in rooms under natural conditions (local temperatures of 15–20 °C, relative humidity 65–70%) for 21 days.

The cheese samples (~250 g) were coated by brushing the coating-forming suspensions on the different cheese faces and left to dry for 1 h at room temperature (15–20 °C). This is the commonly used coating application method for small-scale processes [6]. Two successive coatings were applied once the cheese surface was completely dried. Afterwards, the cheeses were vacuum packaged in polyamide/polyethylene films (thickness 70 μm) and stored at 4 °C for 60 days.

Uncoated cheeses were kept under the same storage conditions than the coated ones and analyzed for comparison. Evaluations of quality attributes were performed at the initial time and after 60 days of storage.

### 2.4. Cheese Characterization 

#### 2.4.1. Proximal Analysis

The proximate analysis of the cheese samples was carried out following standard methods of AOAC: moisture content (926.08), crude protein (2001.11), crude lipid (933.05), and ash (935.42) [17].

#### 2.4.2. Color Attributes

Color was measured using a tristimulus Minolta colorimeter (Konica-Minolta CR-10, Japan) and was reported in CIELab parameters (L*, a* and b* values), where L* was used to denote lightness, a* for redness and greenness, and b* for yellowness and blueness. Hue angle values and color differences (ΔE) were calculated using the following equations:

Hue angle = tan^−1^ (b*/a*) (1)

ΔE = [(ΔL*)^2^ + (Δa*)^2^ + (Δb*)^2^]^1/2^ (2)

#### 2.4.3. PH and Water Activity

The water activity (a_w_) was measured using a AquaLab PRE equipment (Decagon Devices Inc., Pullman, WA, USA).

The pH of the cheese samples was assessed using a HANNA HI5521 digital pH meter (Hanna Instruments Inc., Woonsocket, RI, USA). Cheese samples were macerated and measured using the pH electrode.

#### 2.4.4. Texture Profile Analysis

Texture profile analysis (TPA) of cylindrical specimens of Paipa cheese (20 mm × 25 mm) was performed using a Brookfield CTX texture analyzer (Brookfield Engineering Lab, Inc., Middleboro, MA, USA) equipped with a 10 kg load cell. A double-bite compression cycle was carried out with a TA-AACC 36 probe at 5 g trigger, 50% deformation and a speed of 1 mm/s. Each cheese sample was measured at least 10 times. Cheese textural properties (hardness, cohesiveness, gumminess, springiness, and chewiness) were calculated using Texture Pro software (Brookfield Engineering Lab, Inc., Middleboro, MA, USA).

#### 2.4.5. Microbiological Analysis

Microbiological analysis of the cheese samples was carried out as described in a previous work [16]. The determination of mesophilic aerobic bacteria was performed according to ISO 4833-1: 2013 standard [18]. To count molds and yeasts, the assay was carried out according to ISO 21527-1,2: 2008 standard [19]. Colonies were counted and the results were expressed in log colony-forming units per gram (log CFU × g^−1^).

### 2.5. Statistical Analysis

The statistical analysis was performed using Minitab v. 16 statistical software (Minitab Inc., State College, PA, USA). Analysis of variance (ANOVA) and Tukey’s pairwise comparisons were carried out using a level of 95% confidence. The experiments were performed at least in triplicate, and the data were reported as mean ± standard deviation.

## 3. Results and Discussion

### 3.1. Film Characterization

Table 1 shows the properties of the carvacrol/starch edible films. The properties of neat starch films are also shown for comparison.

Starch/carvacrol films showed similar moisture content, water vapor permeability, and swelling (%) as the neat starch films, indicating that the low concentration of carvacrol used did not affect these physical properties of the starch films. Similar results have been reported by other authors for films made of starch isolated from potatoes and other botanical sources [20].

On the other hand, the carvacrol/starch films showed similar transparency as the neat starch ones (Table 1). It has been reported that the addition of essential oils into edible films could promote opacity due to different factors including the differences in the refractive indices of the film’s components, active compound-matrix interactions that decreased the light passing through the film, and the size and concentration of dispersed particles [21]. Therefore, it can be suggested that the carvacrol concentration used did not affect the homogeneity of the starch films nor their refractive index.

Edible films containing carvacrol showed lower water solubility than the neat starch films (Table 1). This behavior could be attributed to the presence of carvacrol, affecting the water solubility of the starch films due to its hydrophobic properties and low water solubility (~830 ppm) [22].

Figure S2 showed the stress-strain curves for starch and carvacrol/starch films. Initially, the stress increased linearly with the strain, and then a non-linear behavior until failure was observed. This viscoelastic behavior is characteristic of thermoplastic starch [15,23]. Carvacrol/starch films showed a significant decrease in both Young’s modulus and tensile strength in comparison with the starch ones. A similar behavior was reported by Altiok et al. when worked with chitosan films incorporated with thyme oil [24]. In contrast, the strain at break of the films containing carvacrol was higher in comparison with the films without the active ingredient. This increase in the strain at break might be attributed to carvacrol, acting as plasticizer in the starch matrix [25].

The antioxidant activity of starch and carvacrol/starch films was analyzed in terms of radical scavenging ability (Table 1). As expected, the starch films did not exhibit DPPH-scavenging activity, while the carvacrol/starch films showed a DPPH radical inhibition percentage of ~26. The antioxidant activity of carvacrol/polymer films has been reported in previous studies [26,27,28]. It has been suggested that the DPPH-scavenging activity of carvacrol (a monoterpenic phenol) is linked to the steric and electronic effect of its ring, besides the presence of the hydroxyl group, which is capable of donating hydrogen atoms [29,30].

### 3.2. Effect of Edible Coatings on Paipa Cheeses

The initial water content of the cheeses was 35.5 ± 0.7 g water/100 g cheese, while the percentages (*w*/*w*) of fat, protein and ash were 26.8 ± 0.7, 27.1 ± 0.5, and 4.8 ± 0.2, respectively. Taking their water and fat contents into account, these cheeses can be classified as ‘medium fat hard cheeses’ according to codex standard.

**Figure 1:**
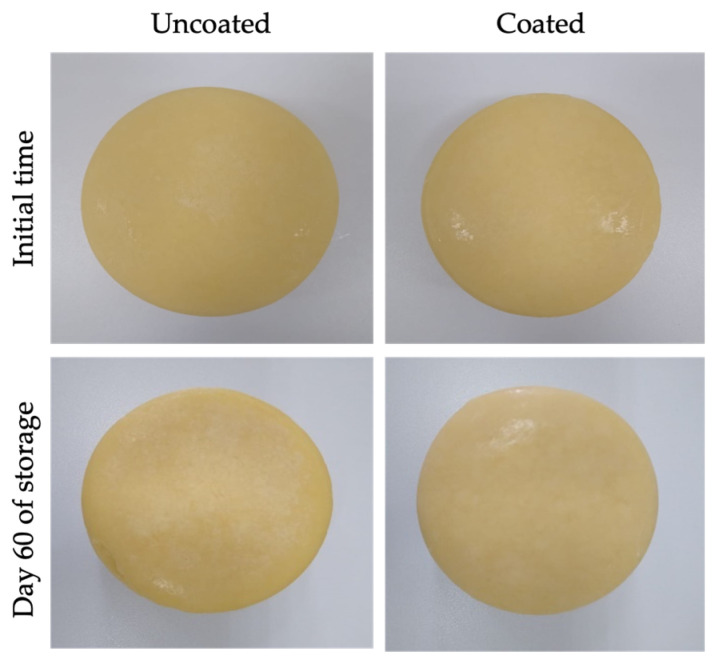
Images of the external appearance of uncoated and coated Paipa cheese samples at the initial time and after 60 days of refrigerated storage.

Figure 1 shows images of the external appearance of uncoated and coated Paipa cheese samples at the initial time and after 60 days of refrigerated storage. It can be noted that the coated Paipa cheese samples were brighter than the uncoated ones. This behavior was attributed to the smoother surface of the coated cheese samples causing a greater reflection of visible light compared with the uncoated cheese surface.

Besides, at 60-days of storage, it was not visualized the presence of fungal colonies in the surface of both uncoated and coated samples (Figure 1).

Table 2 shows the CIELab parameters (L*, a*, and b* values) of uncoated and coated Paipa cheese samples. At the initial time and after 60 days of storage, color differences (ΔE) between uncoated and coated cheese samples were around 1.4 (Table 2). A value of ΔE = 3 has been suggested as an absolute color discrimination threshold for cheeses [31]. In this sense, it can be suggested that the application of the edible coatings did not cause significant changes in the color attributes of the Paipa Cheese.

Uncoated samples showed an increase in their lightness (L*) during the storage (i.e., an increased brightness); whereas, in the coated ones, this color attribute was maintained until the end of the storage (Table 2). Besides, both samples showed a significant increase in the a* coordinate, whereas the b* coordinate values were maintained over time (Table 2). At the beginning of the storage, uncoated and coated Paipa cheese samples showed values of hue angle of 81.9 and 81.5, respectively. After 60 days of storage, the two samples showed a decrease in this parameter, showing values of hue angle of 73.4 and 75.1, respectively (Table 2).

Uncoated and coated Paipa cheese samples showed similar moisture content (~31%) and water activity (~0.95). These parameters were constant throughout the storage, without significant differences between uncoated and coated cheeses (Table 3). It is well known that foods with a high water activity (a_w_ > 0.6) and moisture content are very susceptible to microbial spoilage and contamination [32].

The pH of the Paipa cheese samples ranged from 5.1 to 5.5 (Table 3). At the beginning of the storage, the coated cheese samples showed higher pH than the uncoated samples (Table 3). This behavior was attributed to the that the coating forming solution has higher pH (pH = 5.6) than the uncoated cheese (pH = 5.3). Besides, uncoated Paipa cheese samples showed an increase in the pH during storage, whereas, in the coated samples the pH was maintained over time. Similar behavior was reported by Martins et al. [33] when studied the effect of galactomannans coatings incorporating nisin on the shelf life extension of ricotta cheeses. The increase in pH of the Ricotta cheese was attributed to the liberation of alkaline compounds during proteolysis due to the presence of microorganisms at the cheese surface. Moreover, it was suggested that the slight effect of the coating retarding pH increase over time may be due to the gas barrier properties of the coating helping to prevent the occurrence of cheese proteolysis.

**Figure 2:**
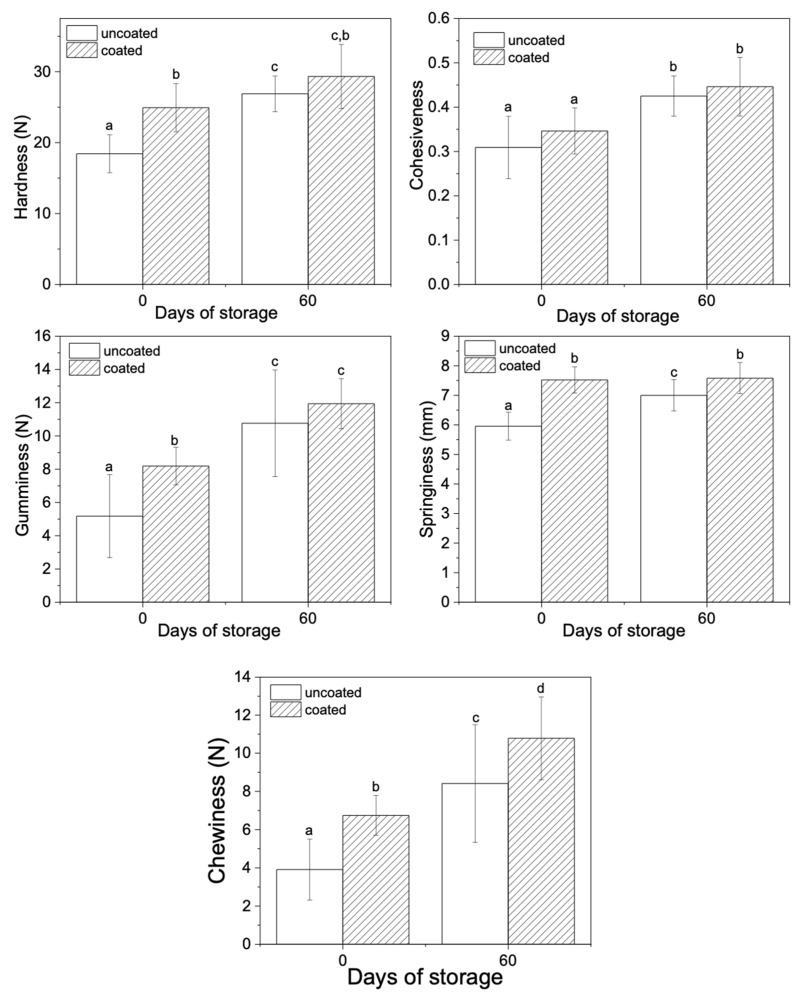
Parameters derived from the texture profile analysis of the uncoated and coated Paipa cheese samples for the initial time and day 60 of storage. Bars with different letters (a, b, c, d) showed statistical difference (*p* < 0.05).

Figure 2 shows the parameters derived from the texture profile analysis of the uncoated and coated Paipa cheese samples. Edible coatings have a significant effect on the hardness, the gumminess, the springiness, and the chewiness of the Paipa cheese (*p* < 0.05), while the cohesiveness of the coated cheese samples was similar to the uncoated ones (*p* > 0.05).

At the initial time, the coated cheese samples showed higher hardness than the uncoated samples (*p* < 0.05) (Figure 2). Cheese hardness has been directly linked to both its bulk and rind consistency [34,35]. Therefore, it could be suggested that, at the initial time, the presence of the edible coating probably affected the rind consistency increasing the cheese hardness. Pieretti et al. studied the effect of the application of alginate edible coatings with oregano and rosemary essential oils on the texture of fresh cheese. At the beginning of the storage, they found that the uncoated sample presented the lowest hardness, proving to be softer than the coated cheese samples [36].

During storage, the uncoated samples showed an increase in their hardness, whereas the hardness was preserved over time in the coated samples. At 60 days of storage, both uncoated and coated cheese samples showed similar hardness. The increase in hardness over time could be attributed to the increase in protein–protein interactions [37].

The cohesiveness of the cheese samples with and without edible coating was similar at the beginning of the storage (*p* > 0.05) (Figure 2). Then, both samples showed a significant increase in this parameter over time, reaching similar values at 60 days of storage (Figure 2).

At the initial time, the coated cheese samples showed higher gumminess than the uncoated samples (*p* < 0.05). This gumminess increase is agreed with the increase in hardness. During storage, both samples showed a significant increase in gumminess. At 60 days of storage, both uncoated and coated cheese samples showed similar gumminess.

At the beginning of the storage, uncoated samples showed lower springiness than the coated ones. This behavior could be attributed to the increase in the pH caused by the presence of the coating [38]. Besides, the uncoated samples showed an increase in springiness during storage, whereas springiness was maintained over time in the coated cheese samples. Several authors have reported that cheese springiness could be increased due to the relaxation of the protein chain caused by proteolysis [9,39].

At the initial time, coated cheese samples showed higher chewiness than uncoated samples. From a sensory point of view, this behavior could indicate that the cheese with edible coatings will require more energy to chew and ingest. Both samples (coated and uncoated Paipa cheese samples) showed an increase in chewiness during storage. These results are agreed with the increase in the hardness and gumminess over time. Similar observations were reported for Gouda cheese coated with glycerol-plasticized starch edible coating carrying antimicrobial agents (natamycin and nisin) [9].

The count of total mesophilic aerobic bacteria of the Paipa cheese samples with and without edible coatings stored at 4 °C for 60 days is shown in Table 4. All samples showed similar bacteria count at the initial time and during storage, regardless of the presence of carvacrol/starch edible coatings. The increase in the bacteria growth over time was around 1.7 log cycle (Table 4).

At the initial time, the application of edible coating caused a slight decrease in the yeasts/molds growth of the cheese samples (Table 4); however, it was not found statically significant (*p* > 0.05). Then, both samples show an increase in the yeasts/molds growth over time reaching similar values at end of the storage. This behavior could be attributed to a burst release of carvacrol from the edible coating at the initial time, decreasing its activity on the cheese surface over time [40,41].

Several authors have reported that some psychrophilic bacteria and molds and yeasts are especially sensitive to carvacrol [42]. However, it has been reported that the antimicrobial activity of edible coatings carrying carvacrol is dependent on its concentration [40,43]. Artiga-Artigas et al. studied the antimicrobial effectiveness of coating containing different concentrations (1.5%, 2.0%, or 2.5% *w*/*w*) of oregano essential oil (a carvacrol-rich essential oil) on low-fat cut cheese. It was found that the coatings carrying oregano essential oil at 1.5% *w*/*w* were not effective in reducing the *Staphylococcus aureus* population, while the use of a greater amount of the antimicrobial agent allows decreasing the microbial population during 15 days of refrigerated storage [42].

A microbiological limit of acceptability = 7 log CFU × g^−1^ has been suggested for cheeses [44]. Thus, both uncoated and coated Paipa cheeses can be considered safe for consumption, and their shelf life was not limited by mesophilic aerobic bacteria and molds/yeasts count during the entire period of storage.

## 4. Conclusions

The application of starch edible coatings carrying carvacrol on Paipa cheese surface probed to be a useful alternative to improve the appearance and to prevent textural changes of the product during storage. The low concentrations of carvacrol used were useful to obtain coated cheeses with similar physicochemical as the uncoated artisanal cheese. Thus, these coatings may act as an additional hurdle to supplement the benefits of the refrigerated storage helping in the maintenance of color attributes, pH, hardness, and springiness.

However, the carvacrol/starch coatings were not effective to reduce the count of total mesophilic aerobic bacteria and molds/yeasts of the Paipa cheese during storage at 4 °C for 60 days. Therefore, further studies are necessary to increase the carvacrol content in edible coatings to obtain a greater antimicrobial action.

## Figures and Tables

**Table 1:** Properties of starch and carvacrol/starch edible films.

Parameters	Starch	Carvacrol/Starch
Moisture content (%)	19.2 ± 1.5 ^a^	20.6 ± 0.9 ^a^
Water vapor permeability (×10^−10^ g s^−1^ m^−1^ Pa^−1^)	4.9 ± 0.2 ^a^	4.9 ± 0.8 ^a^
Transparency	11.4 ± 0.3 ^a^	10.9 ± 0.9 ^a^
Water solubility (%)	23.7 ± 1.2 ^a^	19.7 ± 1.1 ^b^
Swelling (%)	51.9 ± 6.4 ^a^	52.2 ± 4.1 ^a^
Tensile strength (MPa)	17.7 ± 0.5 ^a^	15.3 ± 0.4 ^b^
Strain at break (%)	23.7 ± 3.1 ^a^	33.4 ± 2.0 ^b^
Young’s modulus (MPa)	6.5 ± 0.1 ^a^	2.9 ± 0.6 ^b^
DPPH-scavenging activity (inhibition %)	-	25.8 ± 1.7

Different superscript letters (a, b) within the same row indicate statistically significant differences (*p* < 0.05).

**Table 2:** Color attributes of uncoated and coated Paipa cheese samples.

Parameters	Sample	Day 0	Day 60
L*	Uncoated	66.7 ± 1.1 ^a^	68.3 ± 0.6 ^b^
	Coated	67.9 ± 1.5 ^a^	67.9 ± 1.5 ^a^
a*	Uncoated	3.7 ± 0.3 ^a^	7.7 ± 0.1 ^b^
	Coated	3.9 ± 0.2 ^a^	6.7 ± 0.2 ^b^
b*	Uncoated	26.4 ± 1.0 ^a^	26.0 ± 1.4 ^a^
	Coated	26.0 ± 1.0 ^a^	25.1 ± 0.8 ^a^
Hue angle	Uncoated	81.9 ± 0.8 ^a^	73.4 ± 0.8 ^b^
	Coated	81.5 ± 0.6 ^a^	75.1 ± 0.7 ^b^
ΔE	Uncoated	1.3	1.5
	Coated		

Different superscript letters (a, b) within the same row indicate statistically significant differences (*p* < 0.05). Values of ΔE were calculated to compare uncoated and coated Paipa cheese samples.

**Table 3:** Moisture content, water activity, and pH of uncoated and coated Paipa cheese samples.

Sample	Days of Storage	Moisture Content (%)	Water Activity (a_w_)	PH
Uncoated	0	32.0 ± 3.0 ^a^	0.95 ± 0.01 ^a^	5.12 ± 0.01 ^a^
	60	30.3 ± 1.6 ^a^	0.96 ± 0.02 ^a^	5.31 ± 0.01 ^b^
Coated	0	31.7 ± 1.8 ^a^	0.95 ± 0.01 ^a^	5.45 ± 0.02 ^c^
	60	31.0 ± 1.2 ^a^	0.95 ± 0.03 ^a^	5.45 ± 0.01 ^c^

Different superscript letters (a–c) within the same column indicate statistically significant differences (*p* < 0.05).

**Table 4:** Count of mesophilic aerobic bacteria and molds/yeasts on uncoated and coated Paipa cheese samples at the initial time and day 60 of storage.

Days of Storage	Total Mesophilic Aerobic Bacteria (log CFU × g^−1^)		Yeast and Molds (log CFU × g^−1^)	
	Uncoated	Coated	Uncoated	Coated
0	3.0 ± 0.3 ^a^	3.0 ± 0.2 ^a^	3.3 ± 0.3 ^a^	3.0 ± 0.2 ^a^
60	4.7 ± 0.1 ^b^	4.8 ± 0.3 ^b^	3.8 ± 0.2 ^b^	3.9 ± 0.1 ^b^

Different superscript letters (a, b) within the same column indicate statistically significant differences (*p* < 0.05).

## Data Availability

The data presented in this study are available on request from the corresponding author.

## Supplementary Materials

The following are available online at https://www.mdpi.com/article/10.3390/polym13152516/s1, Figure S1: SEM image of the starch granules isolated from isolated from fresh potato tubers variety Diacol Capiro, Figure S2: Stress-strain curves for Starch and Starch/Carvacrol films, Table S1: Characteristics of the starches isolated from potato tubers variety Diacol Capiro.
