# Supplementary material for: Feasibility of Using Carvacrol/Starch Edible Coatings to Improve the Quality of Paipa Cheese
Source: Polymers (Basel). 2021 Jul 30;13(15):2516. doi: 10.3390/polym13152516 (PMC8347108; doi:10.3390/polym13152516)
Supplement: Supplementary file 1 [file polymers-13-02516-s001.zip › polymers-1292114-SI.pdf]

Supplementary material

Feasibility of Using Carvacrol/Starch Edible Coatings to Improve the Quality of Paipa Cheese

Alex López Córdoba *

Grupo de Investigación en Bioeconomía y Sostenibilidad Agroalimentaria. Universidad Pedagógica y Tecnológica de Colombia. Facultad Seccional Duitama. Escuela de Administración de Empresas Agropecuarias. Carrera18 con Calle 22 Duitama, Boyacá, Colombia.

* Correspondence: alex.lopez01@uptc.edu.co; Tel.: +57-8 7604100.

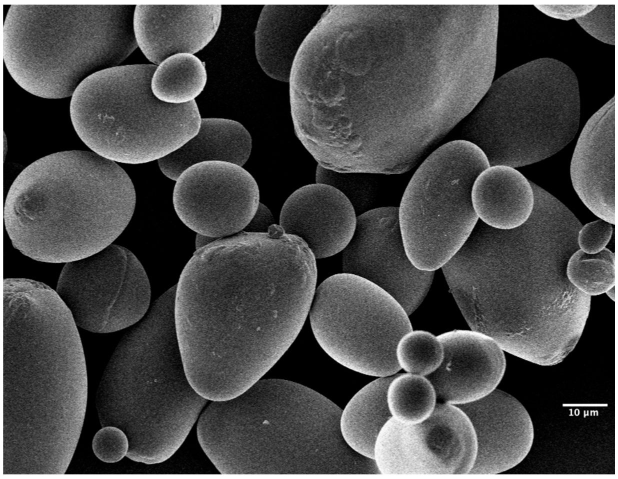

Figure S1. SEM image of the starch granules isolated from isolated from fresh potato tubers variety Diacol Capiro

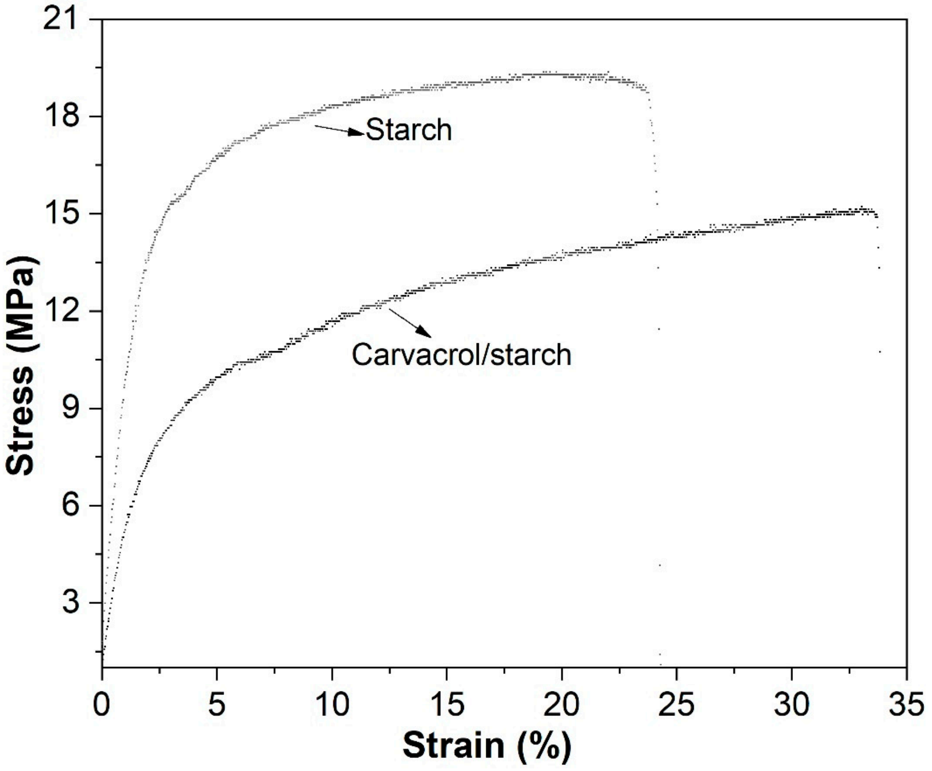

Figure S2. Stress-strain curves for Starch and Starch/Carvacrol films

Table S1. Characteristics of the starches isolated from potato tubers variety Diacol Capiro.

**Characteristic**	**Value**
Extraction Yield (%)	12.0± 1.3
Amylose Content (%)	18.5 ± 1.3
Mean Granule Size (μm)	17.9 ± 0.7
Shape Description	Ellipsoid
Moisture Content (%)	15.2±0.5
